# Community-based participatory research in rural African contexts: Ethico-cultural considerations and lessons from Ghana

**DOI:** 10.1186/s40985-020-00145-2

**Published:** 2020-11-27

**Authors:** Richard Appiah

**Affiliations:** College of Health Sciences, University of Ghana, Accra, Ghana

**Keywords:** Community-based participatory research, Ethical and cultural considerations, Rural Africa, Intervention research, Ghana

## Abstract

Researchers conducting community-based participatory research (CBPR) with vulnerable populations in rural African settings are confronted with distinctive ethical and cultural challenges due to the community context of their research, their methods of investigation, and the implications of their findings for populations. Ethical considerations such as informed consent, the protection of privacy and confidentiality, and relationships between researchers and participants take on greater complexity and have implications beyond the individual research participant. Drawing on careful reflections of experiences from conducting mental health promotion intervention research using the CBPR approach and multi-methods in resource-poor rural communities in Ghana, we examine a range of ethico-cultural issues associated with community-based group intervention research in rural remote settings of Ghana. We offer suggestions to help researchers to envision and manage these complexities in a more appropriate way. Approaches aimed to promote relationships, fairness, respect, and cultural harmony between researchers and study participants are outlined. We urge prospective researchers to carefully explore and respect the cultural values and practices of community members and observe locally-defined ethical values and principles when conducting CBPRs in rural African settings to minimise *ethics dumping* and safeguard the integrity of their research.

## Background

Approximately 60% of the over 1.216 billion people in Africa reside in rural communities [1]. A wealth of research demonstrates that residents of rural, low-resource communities are burdened by distinct experiences and circumstances which are recognisably different in nature and form from those experienced by their urban counterparts. Comparatively, in rural and remote settings in Ghana [2], Kenya [3], Nigeria [4], South Africa [5], and Zambia [6], adults experience higher levels of stress than their urban counterparts and have less access to medical, educational, and social resources [7, 8]. Additionally, these individuals have higher susceptibility to diseases, poverty, and low productivity [9]. Consequently, these constraints blunt their capacity to aspire and to adapt to major traumas, transitions, and adversities, with considerable negative implications for their mental health and economic productivity [10].

There is evidence to suggest that interventions that target health improvements and behaviour modifications of residents of rural poor communities also lead to improved economic outcomes as well, thus yielding a cycle of increasing returns [11]. While the results of these intervention studies can be attributed to the rigorous scientific methods adopted in their designs and implementations, community-based factors, such as the involvement of community members in the research process, and the awareness and adherence to the cultural values and traditional practices of the target population, could also wield significant influence on the research process and the integrity of the findings. Presently, there is scarce literature on on-the-ground field experiences of research conducted in rural, remote, and resource-limited settings in Ghana, and sub-Saharan Africa, more generally. Literature that discusses these experiences can inform and guide the fieldwork plans and processes of future researchers.

In this commentary, we reflect on the process of conducting community-based participatory research (CBPR) across a number of rural, low-resource communities in Ghana. CBPR is an action-oriented research approach that involves a reflective and systematic inquiry in which researchers and community stakeholders collaborate to explore and understand the perspectives and needs of community members with the aims of educating or developing context-appropriate interventions to promote health or cause a behavioural or social change among the target community members [12]. The overarching aim of this commentary is to identify and discuss lessons that may serve as a useful guide for future researchers planning to conduct intervention studies in similar settings. We discuss a range of ethico-cultural issues that pertains to the community entry process; access to households, informed consents, and recruitment; scheduling participants; in-session issues; practicality and cultural sensitivity of session themes; tokens and incentives; recruiting a chief or community elder as participant; dissemination of findings; participation in community activities; and general codes of conducts relevant to intervention research in these contexts.

### Description of projects and field research

The author had the privilege of working on two CBPRs in the rural context of Ghana. The first major field experience involved working as a research implementation associate with Innovations for Poverty Action (IPA) from March 2015 to November 2017. IPA is a research and policy non-profit based in the USA that specialises in conducting the highest quality randomised controlled evaluations of impacts and disseminates outcomes to governments and organisations for policy influence. Working with a senior colleague, we developed a 12-session group-based intervention programme, based on principles and constructs of cognitive-behavioural interventions, for rural poor adults. The two-hour, once-weekly programme was designed to promote participants’ mental health, build their resilience, and increase their ability to take advantage of opportunities and to respond to adverse events. The author led and supervised the recruitment and training of facilitators, piloting of the intervention programme, and the implementation of the scale-up in 165 rural poor communities across four regions of Ghana. Within interactive group discussion and activity sessions, participants were taught about time management, goal setting, problem solving, relationship and communication skills, how to identify and challenge unhelpful thinking patterns, and a host of other behaviour modification and economic empowerment skills. Altogether, 1572 participants were randomly selected across the communities to participate in the sessions. This study forms part of a larger on-going, eight-arm project, the *Escaping Poverty* project [13], that offers a combination of economic package, known as a “graduation programme”, which includes an asset transfer, small cash transfers, access to saving, training, and coaching to participants selected from some 7696 households.

The second project, which is the doctoral research project of the author, also involved developing and evaluating a 10-session multicomponent positive psychology intervention programme (the Inspired Life Programme; ILP). The ILP was developed based on constructs and principles of positive psychology and cognitive-behavioural models and sets out to promote positive mental health and reduce symptoms of depression and negative affect. The novel ILP was evaluated in a sample of adults drawn from four rural poor communities in the middle belt of Ghana, within a quasi-randomised controlled trial [2]. Participants were of both genders, aged between 18 and 60 years, and were peasant farmers and traders in agricultural products. All participants were also native-Twi speakers and lived in communities governed by an elected chief and his elders in a more collectivistic social setting. The results suggest that the programme was effective in promoting mental health and reducing symptoms of depression of participants.

### Community entry processes

The rich cultural values and traditional practices in most African communities, particularly within the rural context, regulate and shape the social lives of the people [14, 15]. In these settings, these cultural values and traditional practices of the people form an integral part of the research process, particularly the community entry process. In the more collectivistic orientation of the Ghanaian rural context, these traditional cultural practices exert significant influence on the psychosocial and behavioural expressions of the people [14, 16]. Similar to the political arrangements in Western and European settings, all communities in Ghana, as also the case in most sub-Saharan African countries, have some form of political institution or structure—with elected *chiefs* or *elders*—and their council members who govern and uphold the tenets and cultural norms of the communities. In the Ghanaian traditional culture, the chiefs or elders are the gatekeepers to the communities who welcome the research team and grant permission for the research to be conducted in the communities. Since the researcher cannot have direct access to or communicate directly with the chief, the researcher first engages with a community elder or council member who welcomes and informs the research team about the customary gifts required to visit the chief. For communities in southern Ghana, these gifts may include hard liquor, usually between two and four bottles of gin or schnapps, or a calabash of cola nuts for communities in the north. The accompanying elder inspects the ethical approval documentations and asks for details of the study, including the aim, significance, and expected duration. This information is presented to the chief and his council—who may also ask for further details, including the rationale for selecting the community for the research. Speaking through his linguist, the chief welcomes the research team and communicates the values and cultural norms of the community to the researchers, who also make verbal acceptance and affirm their preparedness to observe them. The chief then declares the researchers as citizens of the community for the period of their stay and assigns an elder to provide them with the needed support and to ensure their well-being.

Depending on the nature of the research, the researcher makes a request, through the linguist, to the chief for logistical support, such as access to meeting venues and furnishers. The researcher also makes a request for a collaborative search for an independent mediator who would lead the research team to introduce the study and recruit participants in the households. It should be explained that while this individual must be eloquent and likeable, he (often a male) should not be a community leader who exerts some influence and power over the community members, in order to minimise the likelihood of obligating community members to participate in the study. In very remote settings, the chief may task the town crier (*gong-gong* beater) to announce the presence of the researchers and their aim. While expressing appreciation, the researcher carefully explains that such an arrangement could compel community members to participate—even against their volition.

### Access to households, informed consent, and recruitment

Led by a trained independent mediator, the research team selects the households they wish to recruit from. The selection method is based on the research design, sample size, and sampling technique of the study. When a household is approached, the independent mediator leads the team to meet with the head of the household and introduce the study. After permission is granted, the research team identifies individuals who meet the inclusion criteria and randomly select an individual or more, depending on the study design, from the list of household members. The research team, through the independent mediator, introduces the study to the selected individual and addresses any questions or concerns that are raised. As ethically sanctioned [17], the selected individual is given a week to reflect and to contact the independent mediator for further clarification before finalising their decisions to participate. Considering the cultural norms and the patriarchal orientation of most rural settings in Ghana, it is mandatory that the team also obtains permission from the husband of a married female who is selected for recruitment. The team follows-up after a week to recruit individuals who finalise their decisions and consent to participate. The independent mediator reads the content of the informed consent form, in the native language of the individual to be recruited, or translates each phrase verbatim, if the form was not translated into the native language. Individuals who could not sign the consent form are assisted to thumbprint. It is essential that researchers take the necessary steps to tailor the informed consent procedures to the requirements of the study participants to guide understanding and inform their decisions about participating.

Generally, the process of obtaining written (signed) informed consent can be challenging for researchers working with individuals in rural poor settings and populations with a lower level of literacy. For the most part, the majority of participants may exhibit some hesitation about having to read and sign the consent forms, particularly if it is written in English—primarily because they received little or no formal education. While the receipt of a verbal consent affirms participants’ agreement to participate, it may be necessary, whenever possible, to also obtain a signed or thumb-printed (written) informed consent. Together with the independent mediator, the research team explains, during the informed consent process, that some people may be discomfited and anxious about having to sign on the written consent form, even after they provide a verbal confirmation of their participation—and that individuals who do not wish to sign the forms can be assisted to thumbprint on the forms. It should be further explained that the forms contain all the information about the study, including expectations and possible benefits and that a signed or thumb-printed consent form is also a proof of the researcher-participant agreement, which can be presented to the institutional review board (IRB) that approved the study, when evidence of such agreement is requested from the researcher. Researchers—through the independent mediator—should have this discussion with participants at the initial stage of the informed consent process to allay plausible anxiety that may be associated with the signing or thumb-printing on the written informed consent forms. Nonetheless, an initial pilot survey can inform the research team about the potential difficulties that may fraught securing participants’ signatories (or thumbprint) on the written informed consent forms and therefore justify the adoption of verbal consent-only in their protocol to the IRB. To the extent that the majority of participants may have previously participated in a district or national election that involved the thumbprint of ballot forms, the thumbprint option may be a preferred option for most participants and researchers should discuss this as an alternative to signing.

### Scheduling participants

As often the case, the majority of individuals in remote settings who participate in intervention studies are usually farmers—with little or more time at hand, depending on whether or not the research activities coincide with the farming season. Whichever the case, the research team plans a first meeting with the participants to deliberate and decide on convenient days and time for the interviews and the intervention sessions. Participants should be allowed adequate time to exhaust this discussion to reach consensus. This will encourage group cohesion from the start and maximise compliance and retention. The research team prompts participants to consider special days, such as market days, where community members travel to nearby peri-urban communities to trade, as well as other days designated for funerals and other social events. For communities where they exist, participants may settle on *resting* (or sacred) days, when community members take a break from any form of work and rest at home. It is common to have younger participants stay aloof and withdrawn from these discussions, out of respect for their elders. To prevent this, the researcher should facilitate the discussion, albeit passively, and have each individual take their turn to share their views.

### In-session issues

For most participants, partaking in the group-based intervention programme may be the first opportunity to engage in a formal group discussion. This has several implications. First, the majority of participants may be passive and non-interactive—while some others may be overly assertive and domineering. A possible way to minimise this is to lead participants to set group rules to guide the meetings, at the start of the intervention. It should be a shared value for members to allow each other the opportunity to take their turn to contribute to the discussions. Other salient issues such as prompt attendance to sessions, refraining from engaging in side discussions at sessions, and completing homework assignments should be discussed. Second, in rural Ghana, as also seen in most patriarchal societies in Africa, females are often passive—and sadly, are expected to contribute less in discussions that also include men. In highly patriarchal cultures, the researcher should consider holding separate sessions for the genders, in order to increase the interactivity and participation of the female participants. We observe that the high level of religious pluralism and interfaith coexistence in Ghana [18] permit the formation of groups with individuals from different religious backgrounds *without* adverse implications on the participation and interactivity of the group sessions. Third, since group-based intervention sessions are interactive in nature, where participants share their views on themes under discussion, often citing personal example situations, it is important that the research team also discusses the issue of confidentiality with participants before each session commences. Researchers should regularly remind and encourage participants to keep all personal examples and information shared by members as confidential, by explaining the possible implications of a breach of confidentiality on the progress of the sessions. Fourth, before commencing the study, the research team should explore all relevant cultural values and norms of the group and endeavour to adhere to them. In the rural context of Ghana, for example, male researchers (or participants) are not permitted to have physical contact with female Muslim participants, irrespective of the religious affiliation of the male researcher or participant. Overall, it is important that researchers facilitate a discussion on group rules and norms with participants to discuss the above-listed and other salient issues such as prompt attendance to sessions, giving opportunities to others to contribute, and completing home assigned work.

### Practicality and cultural sensitivity of session themes

The majority of individual residents of rural poor communities in Ghana had had no formal education and may be unable to communicate in the English language [18]. Researchers working with this group should make an extra effort to design their intervention sessions to be practicable and interactive and delivered in the native language of participants, since only a few participants may be able to take notes at sessions. One possible way of ensuring this is to involve participants in the formulation process of the intervention programme. In the formative phase, in spite of the framework adopted to develop the intervention programme, a sample of community members from the target population could be recruited to appraise the intervention sessions, within a focus group discussion, and suggest practicable examples that can easily be understood by participants. This is particularly important for interventions that set out to promote mental health, change behaviour, or promote the uptake of a new health programme. Newly developed or adapted interventions should also consider the cultural practices and values of the target population and should avoid words or phrases that may be considered offensive or provocative in that context. In most Ghanaian rural settings, people refrain from citing themselves in examples that involve misfortunes, or death. It is acceptable, for example, to say “When someone passes on…”, than to make a direct reference, such as, “When I die…” or “When you die…”. In intervention sessions that seek to identify human strengths, for instance, some participants may find it problematic to discuss about their personal achievements or material resources in the public space, as they may consider such self-evaluations as *being* boastful, which could lead to intrapsychic conflicts when enforced [14, 19]. A good example of a Ghanaian proverb that succinctly teaches about reticence says, “Showing off made the hyrax lose its tail.”

Another way to safeguard the practicality of intervention sessions for rural participants with little or no formal education is to structure the intervention sessions to include mini-lectures, focused group discussions, demonstrations, and brief exercises—with several breakout sessions. Session facilitators can write the session themes, goals, and outlines in the native language and display on a flip chart at each session. Essentially, sessions should be designed to stimulate interactive discussions where participants take turns to share their views and ask questions. Typically, each intervention session can be divided into three parts. The first part could focus on a review of the previous session and a discussion of home assignments and feedback on the application of previous lessons and skills. The second part may focus on explicating the current theme and content, using breakout activities and exercise sessions. The last part may be used to review key lessons and skills, discuss homework assignments, and provide a brief introduction of the next session.

A methodological strategy to drive the therapeutic process of group-based intervention programmes in this context could be to adopt a practical implementation approach that communicates the lessons to participants in an effective fashion. In a recent quasi-randomised controlled trial, researchers utilised several practical exercises to teach participants about goal setting skills, time management skills, problem-solving skills, relationship and communication, personal growth, self-acceptance, and a host of mental health and life improvement skills to a sample of adults recruited from four rural poor communities in middle Ghana [2]. In teaching about time management and goal setting skills using the empty jar demonstration, session facilitators tasked two volunteer participants to pour a quantity of small rocks, pebbles, and sand into a transparent glass jar. The level of the content is noted. The pattern of arrangement is reversed, using the same quantity of materials in the same jar—this time the jar was filled to the brim. Participants were tasked, in a breakout session, to answer a set of questions about the demonstration and discuss how they can apply the lessons from the exercise. Three months after the intervention, participants could vividly recall the exercise and discuss its significance and how they were applying them to enhance their vocational productivity [20]. Results from the main programme evaluation also showed that the intervention programme was effective in promoting mental health and reducing symptoms of psychopathology in the participants [2].

### Tokens and incentives

As appreciation for their time and effort in the study, it is ethically mandated that participants are provided with some tokens or incentives [21]. IRBs ensure that the value and mode of presentations of tokens and incentives that researchers present to study participants do not influence their decisions to participate in the study, or exploit them. Contexts matter: Would a tin of milk influence the decision of participants from an urban, industrialised setting to participate or alter their responses? Would this be different if the participants are from a remote, rural poor setting? The researcher’s decision about the value and form of incentives is guided by approvals from the IRBs, who usually prescribe a standardised rate for incentivising participants. Before submitting a protocol for ethical approvals, researchers should endeavour to enquire about the form and value of tokens from previous researchers from the setting, district assemblies, or analyse data collated on the living standards of the target population to guide their determination and justify them in their protocol to the IRB. On the field, researchers should withhold discussions about incentives or tokens at the beginning of the interviews or intervention sessions. If possible, tokens should be hidden in field backpacks and out of sight from the participants. It is also important to emphasise to participants that incentives are given to express appreciation for their time in the study and not for their responses to questions in the interviews or their contributions in the intervention. While not necessary, researchers can also vary the gifts presented, particularly for participants who participate in a 5- or 10-session intervention programme that spans several weeks. Gifts such as bar of soap, box of sugar, canned drink, or exercise books and pencils (for their school going children) may be presented in alteration, weekly. It is a common occurrence for participants from these collectivistic rural settings to show appreciation to the session facilitators for selecting them into the intervention programme or for the lessons and skills they are being taught in the programme, by also presenting gifts such as baskets of fruits, tubers of yam, and chickens to the research team. The researcher shows appreciation for the gift and carefully explains that it is against their work ethics to receive such gifts from study participants. Of note, parting gifts from participants may be accepted when the study is completed or on the day of departure from the community. Among some ethnic groups, a visitor’s rejection of a parting gift could be interpreted as disrespectful or discourteous. To avoid such complexity, the researcher should discuss about gift sharing with participants at the beginning of the study.

### A chief or community elder as participant

Field surveys and baseline assessments often precede the implementation of intervention programmes. Given that the entire population of small, rural, and remote communities may be surveyed, it is possible that the chief or his elders may be recruited into the intervention group, if a subset of the previously surveyed population is randomly selected for the intervention programme. There are several implications when a chief or community elder is selected to participate in an intervention programme. First, the presence of the chief or elder could inspire participants to be punctual to sessions, out of respect for the chief or elder. However, his participation could also lead to lack of interactivity and monotony. In the highly patriarchal rural context of Ghana, younger individuals are more likely to refrain from offering counter suggestions to those postulated by an elder—more so if they were made by the chief. In the unlikely event that a chief or community elder is randomly selected to participate in a group-based intervention programme, the research team, together with the independent mediator, may visit the “participant” to congratulate him for his selection. Carefully, the team enlightens him about the implications of his presence and solicits for his views to minimise the potential influence of his presence on the session. The team adds that he may choose to sit at the back of the session, speak last, build on points made by members, give commendatory remarks to members’ comments, and exercise caution with his remarks in order not to appear judgemental of members’ suggestions.

Although the chief and elders of the community *do* weigh more influence over the community members than do the independent mediators (who were carefully selected to minimise the possibility of coercing participants to participate), the research team is ethically obligated to include the chiefs and elders in the study once they are randomly selected into the study (i.e., after their initial inclusion in the baseline survey). In small, rural remote settings, the chiefs and elders frequently encounter and interact on daily basis with the community members. To this end, their participation in the group sessions may have minimal effect on the session, compared to their counterparts from relatively *larger* rural, peri-urban, or urban communities where community members do not have frequent encounters with their chiefs or elders. Furthermore, an independent mediator with an authority role in the community who leads the recruitment process may likely influence and coerce all individuals he approaches to agree to participate in the study—with a greater ethical implication, compared to a chief or elder selected into a group where the above suggested protocols are observed to minimise the potential influence of his participation on the intervention programme. We recommend that researchers planning to recruit samples from several *large* rural or peri-urban communities should consider excluding the chiefs and elders from their research and explain this decision in their protocols to the IRB.

### Dissemination of research findings

It is likely that the research team may exit the communities once the intervention and data gathering have been completed—before the data is analysed. Nonetheless, researchers are ethically obligated to share their findings with participants and communities involved in the study after the data is analysed [22]. Dissemination of results should be included in the research plan so participants get to know the outcome of their involvement and contribution and to encourage them to participate in future studies. As soon as the study results are analysed and interpreted, the researcher conducts “pre-meetings” with gatekeepers (i.e., participants, representatives from the district or regional health agencies, independent mediator, a chief or a delegated elder). Researchers explain their findings to participants and have them confirm if the main findings represent their views, explore the possible relevance of the findings for community members and the interested (health) agencies, and to brainstorm the best strategy to present the findings to the entire community. When the chief was not present at the pre-meeting, a delegation that includes the researcher may visit the chief and his elders to inform them about the findings and the planned dissemination and jointly schedule a convenient time for the dissemination of the findings. When necessary, the team solicits for the chief’s support to organise logistics, such as meeting space and furnishers for the event. The chief is also informed about other guests, such as members of the district health bodies or regional departments (e.g., District or Regional Health/Mental Health Coordinators) who might be interested in or compelled by the research findings. The team prepares a PowerPoint presentation in the native language of participants, with large fonts and photographs, to identify and explain themes, discuss findings, clarify misconceptions, and increase participants’ understanding and knowledge about any of the study’s findings. To facilitate participation from the audience and to substantiate their understanding of the information presented, an interactive questions and answers (Q&A) strategy can also be used. Using non-technical terminologies, the researcher emphasises and assures study participants and members of the community about the security of the data, the anonymity of the participants in study publications, and the measures instituted to ensure the confidentiality of the data.

### Participation in community activities 

For the most part, researchers are accorded the same privilege and often considered as members of the community during their period of stay. To this end, researchers may be invited by participants or the chief to attend social events, such as funeral rites, church services, or the naming of a baby. Group members may also plan to visit a member who is ill or bereaved. When possible, the researcher may honour these invitations. It is mandatory, however, if the invitation comes from the chief. Although it is not obligatory, the research team can plan a visit to the chief’s palace and provide a brief update of the progress of the intervention, particularly if the session runs for several weeks.

Individuals in rural, collectivistic settings value their relationships and accord respect to visitors who work to improve the well-being of community members. It is possible for participants or chief to call up members of the research team to enquire about their well-being several weeks or months after the study has been completed. This is often the case if the researcher developed a trusting and honest relationship with participants. In the Ghanaian rural settings, relationships do not die—they flourish.

### Code of conducts

Conducting empirically sound research in rural African contexts goes beyond securing ethical approvals from the IRB and permits from relevant agencies. It is important that researchers, from local and foreign institutions, strive to observe all applicable code of conducts in the local context of their study to avoid ethics dumping—the practice of exporting unethical research practices to lower-income settings. Researchers should acknowledge that codes of conduct and values such as fairness, respect, care, and honesty take on greater complexity in low-income, resource-poor settings. For instance, it is imperative to determine, within collaborative partnerships with local partners, about the local relevance and feasibility of the proposed research. Research with little or no local relevance only imposes undue burden on the participants without any benefits [23]. Where possible, the target communities and research participants should be included in the entire research process to enhance participation and the intended impact.

To safeguard the integrity of the research process and findings, researchers working in rural communities with low literacy populations should endeavour to translate the consent forms into the native language of the target group to facilitate participants' understanding and acceptance of the study. In furtherance, researchers should administer, when available, questionnaires that have been translated and validated in the native language of the people involved to increase their reliability and validity. Given the vast cultural differences that exist between people from different social structures and value orientations, for instance, between Western and African societies [24], research is needed that translates and validates measurement instruments developed from a Western perspective with unrepresentative Western samples (who are more individualistic) into the native languages of the intended target group before they are administered. In a recent effort, researchers in Ghana translated and examined evidence for the factorial validity of the Twi versions of five mental health and well-being measurement instruments, including Affectometer-2, Automatic Thought Questionnaire–Positive, Generalized Self-Efficacy Scale, Patient Health Questionnaire-9, and Satisfaction with Life Scale in a rural Ghanaian adult sample [25].

Since the majority of the supporting staff from the study communities would zealously dedicate their time and resources to support the success of the research project, researchers must exercise diligence and fairness in their compensation of these support staff, such as independent mediators, translators, interpreters, or local coordinators. It is also important for the researcher to keep in mind that research, in all its forms, *is* a voluntary exercise for participants. Under no circumstance should researchers attempt to impose their ethical values on research participants. It is possible that researchers from high-income and urban settings may disagree with local communities and participants on how to conduct the research. However, at all times, the decisions and stance of the local stakeholders and participants surpass those of the researcher. In this regard, researchers must endeavour to negotiate rather than impose their ideologies and perspectives on study participants in rural, resource-poor settings.

## Conclusion

Conducting CBPR in low-resource, rural poor, and remote communities in sub-Saharan Africa is both rewarding and challenging. The researcher is confronted with a distinct set of cultural and ethical issues, which can often be utilised to facilitate the research process. Community members often regard researchers conducting CBPR in their communities as partners and are supportive of the research process. The researcher must first establish a trusting and honest relationship with the gatekeepers (i.e., chiefs and community elders) and local supporting staff, such as independent mediators, who then lead the researcher to engage with participants in ways that are culturally sanctioned. While meandering their ways through these cultural values and traditional practices associated with the various stages of the research, the researcher should also strive to adhere to the scientific principles and methodologies underpinning their research in order to safeguard the integrity of research process and the study results.

## Data Availability

Not applicable.

## Abbreviations

CBPR: Community-Based Participatory Research
ILP: Inspired Life Programme
IPA: Innovations for Poverty Action
IRB: Institutional Review Board
Q&A: Questions and answers
